# A Design of a New Column-Parallel Analog-to-Digital Converter Flash for Monolithic Active Pixel Sensor 

**DOI:** 10.1155/2017/8418042

**Published:** 2017-01-24

**Authors:** Mostafa Chakir, Hicham Akhamal, Hassan Qjidaa

**Affiliations:** CED-ST, LESSI, Faculty of Sciences Dhar el Mehraz, Sidi Mohamed Ben Abdellah University, BP 1796, Fez-Atlas, 30003 Fez, Morocco

## Abstract

The CMOS Monolithic Active Pixel Sensor (MAPS) for the International Linear Collider (ILC) vertex detector (VXD) expresses stringent requirements on their analog readout electronics, specifically on the analog-to-digital converter (ADC). This paper concerns designing and optimizing a new architecture of a low power, high speed, and small-area 4-bit column-parallel ADC Flash. Later in this study, we propose to interpose an S/H block in the converter. This integration of S/H block increases the sensitiveness of the converter to the very small amplitude of the input signal from the sensor and provides a sufficient time to the converter to be able to code the input signal. This ADC is developed in 0.18 *μ*m CMOS process with a pixel pitch of 35 *μ*m. The proposed ADC responds to the constraints of power dissipation, size, and speed for the MAPS composed of a matrix of 64 rows and 48 columns where each column ADC covers a small area of 35 × 336.76 *μ*m^2^. The proposed ADC consumes low power at a 1.8 V supply and 100 MS/s sampling rate with dynamic range of 125 mV. Its DNL and INL are 0.0812/−0.0787 LSB and 0.0811/−0.0787 LSB, respectively. Furthermore, this ADC achieves a high speed more than 5 GHz.

## 1. Introduction

CMOS Monolithic Active Pixel Sensors (MAPS) are charged particle tracking devices, integrating on the same silicon substrate radiation sensitive detector elements with its front end readout electronics. In the past years, the CMOS Monolithic Active Pixel Sensors (MAPS) [1–3] have evolved as an interesting alternative to fulfill the requirements of vertex detector in the future high energy physics and biomedical imaging applications compared to the existing detectors like Charge Coupled Devices (CCD) [4–9] or Hybrid Pixel Detectors (HPD) [10]. The MAPS has many successful advantages such as high spatial resolution, low cost fabrication, low power, radiation hardness, compactness, random access, and fast readout. Nevertheless, despite all these advantages many other challenges still linger for the future of International Linear Collider (ILC) vertex detector (VXD) [11]. There are three steps to deal with the very small amplitude pixel signal (around a couple of millivolts). The first step is the readout chain that must have a low noise limitation, the second is the speed of the readout circuitry that must be fast in order to realize an integration time ranging from 10 to 100 *μ*s, the speed of the readout circuitry must be fast, and the third one is a further decrease in power consumption and active area that is strongly desirable.

The popularity of the column-parallel readout architecture in improving the readout speed, allowing reading up to 10 k frames/s, leads to the designing and the fabrication more than 30 different minimum ionizing particle MOS active pixel sensors (MIMOSA) [12, 13]. Sensors equipping the innermost layer of the ILC VXD must show a single point resolution better than 3 *μ*m attached to a very short integration time (less than 10 *μ*s) because of the beam strahlung background. This prerequisite encourages an R&D effort centring on a high readout speed design. A small pixel pitch of 16 *μ*m (called MIMOSA-30) ended with a discriminator is proposed [14–17]. The largest sensors for the outer layers which stand for about 90% of the total VXD surface appeared to have less confines in terms of spatial resolution and readout speed. To achieve the minimization of the power consumption a single point resolution of 3-4 *μ*m must be combined with an integration time shorter than 100 *μ*s, whereby it is supposed to form a valuable trade-off. A larger pixel pitch of 35 *μ*m combined with a 4-bit ADC is proposed [18, 19], therefore reducing the power consumption without losing the spatial resolution.

The different kinds of ADCs architectures have been studied by several researchers [18, 20–33]. The proposed ADCs architecture determines how well it can meet the below-mentioned targets. In literatures, pipeline [20], double ramp [21], and successive approximation register (SAR) [18, 22, 23] are notable for achieving the needed specifications. Indeed, Dahoumane et al. [20, 24] and Bouvier et al. [25] have proposed that the pipeline architecture can get a high speed; however, it requires several operational amplifiers, which results in a large power dissipation. Pillet et al. in [21] have proposed that the double ramp architecture can get a low power consumption and small area, but it is not suitable for conversion speed of 1 M samples/s. The SAR architecture proposed by Zhang et al. [18, 22, 23] requires several comparisons cycles to complete one conversion and therefore has limited operational speed. In fact, each ADC has its advantages and weaknesses that make it more compatible with different applications. Several key points have been used for a converter design to develop a certain formulae that could compare different architectures and some of these points are the accuracy in bits, power dissipation, the speed conversion, and more [2]. Note that, if the pipeline, double ramp, and successive approximation register (SAR) ADC architectures have been widely used in literature to design the MAPS, no work is interested in the use of Flash ADC architecture where the power consumption of the column ADC is a very critical issue. For this, we have proposed, in this paper, a new architecture 4-bit column-parallel ADC Flash, low power, high speed, and small area. In this proposal, we interpose an S/H block in the converter. This integration of S/H block increases the sensitiveness of the converter to the very small amplitude of the input signal from the sensor (around a couple of millivolts) and provides a sufficient time to the converter to be able to code the input signal. This ADC is developed in a 0.18 *µ*m CMOS process with a pixel pitch of 35 *µ*m. This paper describes the design of a column-parallel ADC suitable for the outer layer CMOS sensors where the design of a new 4-bit column-parallel ADC Flash is constrained by several factors:

- The used technology must be the one already validated to achieve the pixels.
- The ADC needs the conversion of continuous signal and hence does not have dead time.
- In order to accomplish an integration time of 100 *μ*s or less in a full size sensor (about 2 × 2 cm^2^), the ADC accommodating the pixel readout in parallel is required to work at a frequency of 100 MHz (10 ns/row).
- The design of layout has to be adjusted to the dimensions of the pixel (width 35 *µ*m).
- The readout chain should introduce very feeble noise in order to contain the modest pixel signal (around a couple of millivolts).
- The power consumption of ADC must be minimized.

The simulation results of the proposed ADC respond to the constraints of power dissipation, size, and speed for the MAPS sensors compared with Zhang [18, 22, 23], Dahoumane [20, 24], and Bouvier et al. [25]. The ADC must be compacted, fast at a sampling frequency (100 MS/s), very low power dissipation, and responsive to a minimum signal of approximately 7.81 mV. This minimum signal delivered by each column is typically of the order of mV, and it is a first challenge to the design of the read circuit. The choice of this ADC is a compromise between the granularity and the spatial resolution of the sensor, the size, and power dissipation.

## 2. ADC Design

**Figure 1:**
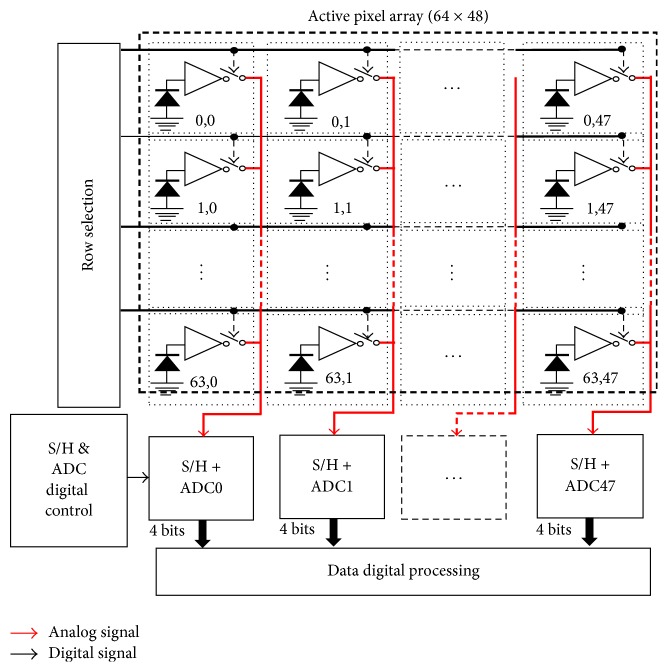
Global architecture of MAPS array access per-column ADC (PC-ADC) and integrated readout electronics.

The global architecture of MAPS chip comprising the pixel array with its associated readout electronics and conversion stages is showed in Figure 1.

Unlike the conventional Flash ADC architecture where the input signal from the sensor is directly linked to the comparators, we propose to interpose an S/H block in the converter. Indeed the integration of an S/H block in the converter will

- increase the sensitiveness of the converter to the very small amplitude of the input signal from the sensor (around a couple of millivolts);
- provide a sufficient time to the converter to be able to code the input signal.

Furthermore, the architecture of this block S/H was optimized using a minimum number of components capable of performing several operations of the conditioning signal.

**Figure 2:**
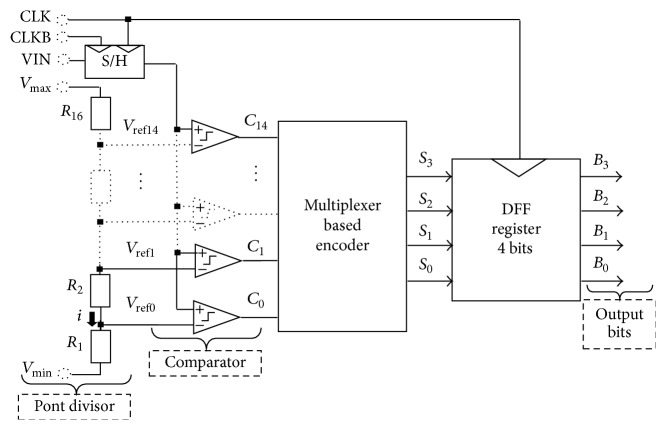
New proposed architecture of ADC Flash.

The ADC converts the pixel output signal by using a new Flash ADC architecture based on a multiplexer based encoder and a specific sample-and-hold (S/H) circuit, as shown in Figure 2. The main components are a sample-and-hold (S/H) circuit, pont divisor circuit, series of comparators circuits, a multiplexer based encoder circuit, and DFF register circuit. A sample and hold (S/H) circuit is employed to sample and amplify the pixel signal. A pont divisor of resistors placed in series generates references voltages of comparators. For the 4-bit converter, we need a ladder with 16 resistors. Here the maximum voltage is divided by 16. The series of comparators composed of 15 comparators including a buffer and a preamplifier are used to adapt the level of the voltage references supplied by the “pont divisor.” The output of a comparator is 1.8 V when the input voltage becomes greater than the concerned voltage reference and 0 V otherwise. A multiplexer based encoder uses 2 : 1 multiplexer requiring 11 multiplexers for implementing 15 inputs and 4 inverters which convert thermometer codes to the binary codes. It should be noted that at this step the output signals are not synchronous. To solve this problem, a DFF register is proposed to allow a synchronous binary signal using four flip-flops of type latch. The output signals are composed of 4 bits that come out in parallel.

### 2.1. Proposed Sample-and-Hold Circuit (S/H)

**Figure 3:**
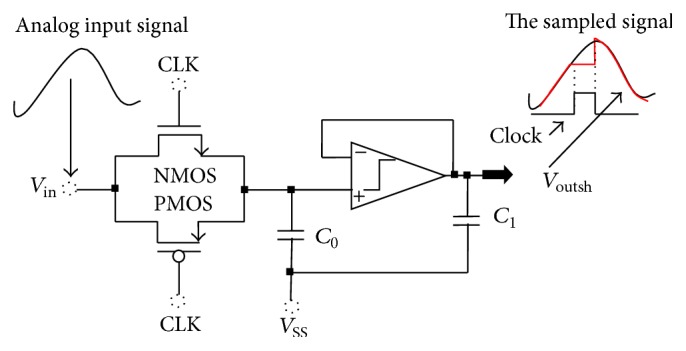
Architecture of a proposed sample-and-hold circuit.

To increase the sensitiveness of the converter to the low amplitude of the input signal from the sensor (around a couple of millivolts) and to provide a sufficient time to the converter to be able to code the input signal, we propose to interpose an S/H block in the converter. As shown in Figure 3, the architecture of the proposed sample-and-hold (S/H) circuit is consisted of an output feedback of operational transconductance amplifier (OTA); a hold capacitor and a switch operate at sampling frequency. The voltage at the terminals of the capacitor follows the voltage to convert when the switch is off, this is on one hand. On the other hand, when the switch is on the voltage at the terminals of the capacitor no longer follows the changes in the frequency of the signal to convert. Transmission gate (TG) is used as a switch and a hold capacitor *C*_0_ value is 250.8 fF. The idea behind using transmission gate (TG) as switch is to get maximum sampling frequency. Generally, upper limit on sampling frequency is depended on the type of a used switch, and with TG as switch, we can get around 6.25 MHz to 5 GHz of sampling frequency without strongly affecting the output. The main advantages of this architecture are the charge injection error and the clock feedthrough error, and they are effectively removed. This type of S/H obtains a very high-accuracy characteristic.

The S/H architecture can get a high speed and low noise performance. The gain of this S/H circuit is

(1) $$\begin{array}{c} G_{S/H}=\frac{V_{outsh}}{V_{in}}\approx A_{v}\times \frac{1}{1+j\omega R_{ON,TG}C_{0}}. \end{array}$$

Here, *A*_*v*_ is the gain of the operational transconductance amplifier (OTA) circuit. The value of *R*_ON,TG_*C*_0_ is chosen to limit the *KT*/*C* noise effect. In order to maximize the gain, *R*_ON,TG_*C*_0_ should be minimized; yet, that would produce a large parasitic capacitor in layout causing great current to drive in the OTA. Consequently, a trade-off must be taken between gain and power.

**Figure 4:**
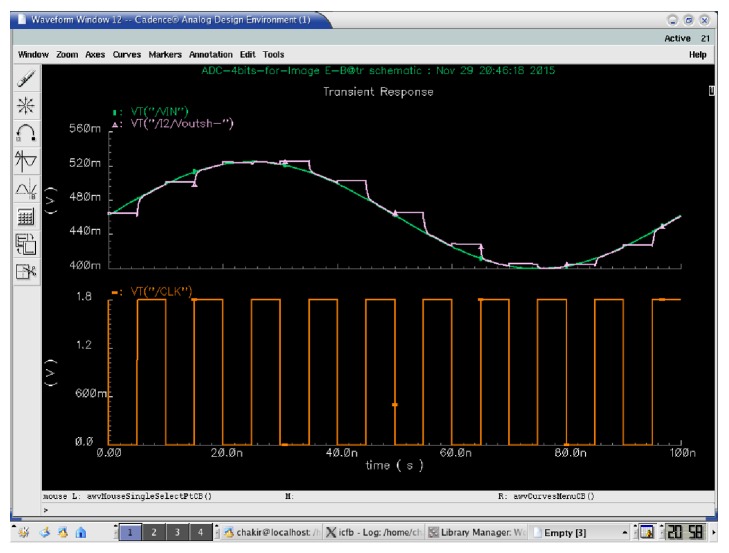
Sample-and-hold output for sampling frequency *F*_S_ = 100 MHz and *F*_in_ = 10 MHz.

Figure 4 shows the simulation of our S/H for an input signal frequency *F*_in_ = 10 MHz and a sampling frequency *F*_S_ = 100 MHz.

#### 2.1.1. Operational Amplifier Circuit

In the sample-and-hold circuit the operational amplifier is very important to get accurate results [34]. We propose the use of an operational transconductance amplifier which has a gain of about 103 dB for a bias current of 9.5 *µ*A with *V*_DD_ = 1.8 V and *V*_SS_ = 0 V. A value of loading capacitor is 0,1 pF. The architecture of the proposed amplifier is composed of three stages: a differential input stage that pilots an active load, a gain stage which increases the gain, and an output stage that can be added for the conduct of large loads off-chip. This configuration offers a good common mode range, a swing of output, the voltage gain, and the Rate of Common Mode Rejection (CMRR) in a simple circuit that can be compensated by a capacitor and resistance.

**Figure 5:**
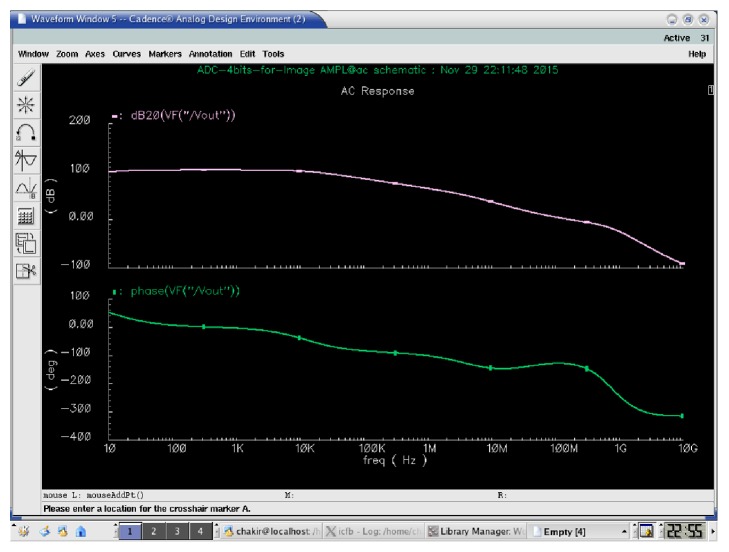
Bode diagram of the proposed OTA.

The performances of operational transconductance amplifier (OTA) for which we have optimized the gain, PM (Phase Margin), GM (Gain Margin), the CMRR (Rate of Common Mode Rejection), PSRR (Power Supply Rejection Ratio), ICMR (Input Common Mode Range), BW (bandwidth), and the power dissipation (Pd) are all showed in Table 1.  Figure 5 shows the open loop gain and phase margin of the proposed OTA.

### 2.2. Comparator Circuit

Among different architectures in literature we chose the architecture of a static comparator [34]. Indeed, it presents the advantage of a low offset and a switching noise that are reduced to a lower input. The configuration of the proposed Comparator Architecture consists of three stages. The first stage is differential input pair, which are PM1 and PM2 of P-channel type, charged by active load of NM1 and NM2 of NMOS transistors and polarized by PM3 transistor. The second stage is added to increase the gain in differential mode. The last stage consists of two inverters (NOT gate) and its role is to achieve a clear switching.

The performances of a comparator which are the open loop gain, the Slew Rat (SR), ICMR (Input common Mode Range), offset, bandwidth, setting time, and the power dissipation are all showed in Table 2.

### 2.3. The Digital Part

The digital part is composed of an encoder and a register. The encoder transcribes data from the comparators stages to the binary signal using the thermometer code. It should be noted that at this step the output signals are not synchronous. To solve this problem, a DFF register is proposed to allow a synchronous binary signal using four flip-flops of type latch. The output signals are composed of 4 bits that come out in parallel.

#### 2.3.1. Proposed Multiplexer Based Encoder Circuit

**Figure 6:**
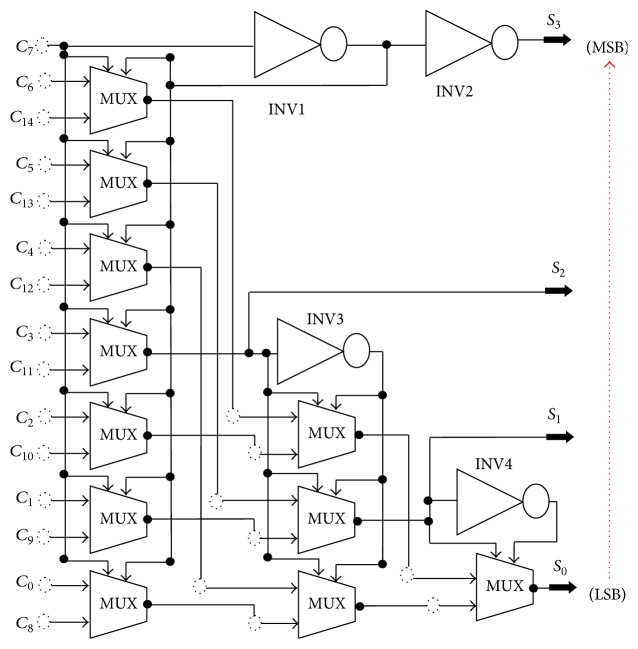
Logic diagram for proposed circuit of multiplexer based encoder.

The several architectures of encoders have been studied by several researchers [35–39] to convert thermometer code to binary code. In the literature, S$\ddot{a}$ll et al. [35] and Wallace [36] have proposed that the Wallace tree based decoder uses the one counter; the output is the decoded binary code and it also applies global bubble error correction/suppression. So, this approach has the benefit of bubble suppression. The disadvantage of this approach is that it results in large delay and power. Lee et al. in [37] have proposed that the Fat tree based decoder architecture can get a low power consumption and delay efficient. However, these results are in reduced area and delay in comparison to Wallace tree based decoder. A more optimized implementation of the Fat tree based encoder is presented by Hiremath and Ren [38]. This approach neither reduces the array of OR gates into NAND-NOR pairs. The NAND-NOR gates were implemented using a pseudodynamic CMOS logic. Sa$\ddot{a}$il and Vesterbacka [39] have proposed another architecture named the existing MUX based decoder; this latter results in short critical path and small area. Nevertheless, this proposed architecture results in huge fan-out in the critical path. Therefore, the increased fan-out causes an increased power consumption and delay. Note that, the Wallace tree based decoder, the Fat tree based decoder, and the existing MUX based decoder architectures have been widely used in the literature to design the ADC. Up to now, no work has been done to improve the multiplexer based encoder architecture where the power consumption is a very critical issue. For this, we proposed a new architecture 4-bit encoder, low power, high speed, and small area. The multiplexer based encoder circuit uses 2 : 1 MUX, so we require 11 MUX for implementing 15 inputs and 4 inverters which convert thermometer codes to binary codes. The 2 : 1 MUX needs two input signals with one select line; the select line should vary between two logics 0 to 1 depending on the select line the MUX 11 transmit the logic. The logic of the most significant bits (MSB) of the binary input is equal to the middle bit of the thermometer code because it follows the twin logic. The logical encoder used for ADC 4 bits is represented in Figure 6. It should be noted that at this step the output signals are not synchronous. To solve this problem, a DFF register is proposed to allow a synchronous binary signal using four flip-flops of type latch. The output signals are composed of 4 bits that come out in parallel.

The truth table for 4-bit multiplexer based encoder is shown in Table 3. The MSB bit of the output is equal to the *C*_7_ bit of input (middle bit) and least significant bit (LSB) of output is equal to the value of *C*_14_ to *C*_0_. In this design 11 multiplexers are used because in first stage there are 15 inputs for implementing 15 inputs: 7 MUX are used in the second stage; 3 MUX are used; the output of middle multiplexer is acting as select line in the second stage while in last stage 1 MUX is required. The multiplexers used are designed using transmission gates for better accuracy.

**Figure 7:**
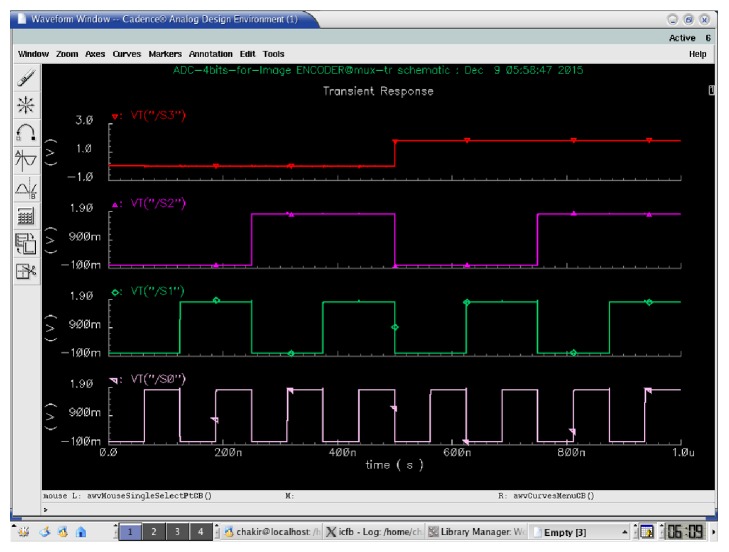
Multiplexer based encoder output.

Figure 7 shows the encoder simulation that allows encoding the thermometers code to the binary code. The outputs of 15 comparators are noted from *C*_0_ to *C*_14_ and the outputs of binary code are *S*_3_, *S*_2_, *S*_1_, and *S*_0_.

#### 2.3.2. TG-Register Circuit Based on *D*-Type Flip-Flop

**Figure 8:**
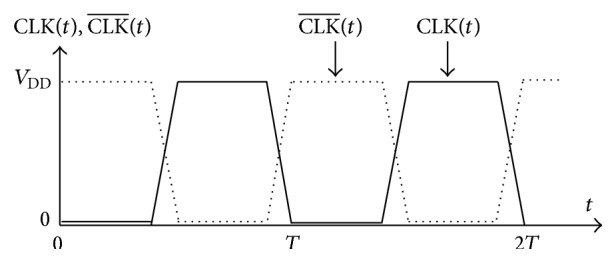
TG clocking signals.

The clock signal CLK(*t*) is applied in different CMOS circuits for their operations.  Figure 8 shows the clock signal CLK(*t*) and its complement $\overset{-}{CLK}(t)$. The synchronization of the operations in a digital network is performed by means of signal of clock with respect to an absolute time base. The period, denoted *T*, is the time interval per unit time in seconds, which corresponds to the inverse of the period: *f* = 1/*T* or *f* is the frequency in Hertz (Hz or s^−1^). The complement signal of CLK(*t*) is denoted as $\overset{-}{CLK}(t)$. The synchronization of the data flow is performed by the clock signal when the TG may be activated or deactivated with a complementary pair [40].

**Figure 9:**
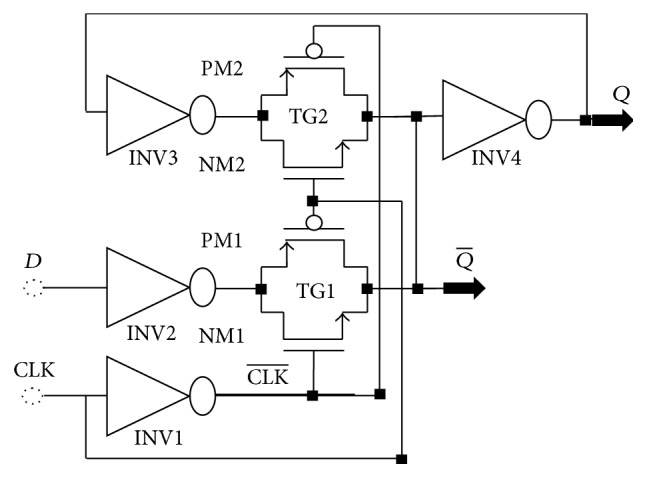
Basic TG-latch circuit.

**Figure 10:**
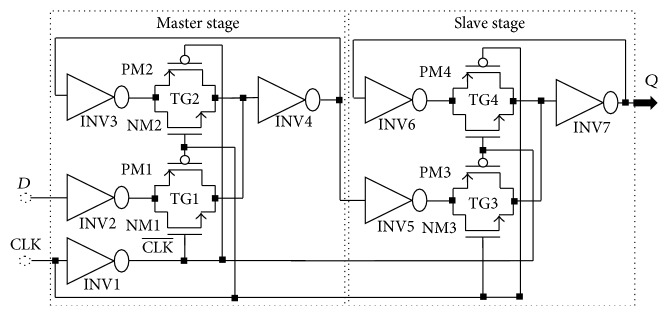
Diagram TG-Register based master-slave flip-flop.

Here, our idea is to create a low power TG-Register circuit *D*-type flip-flop (Master-slave) in CMOS technology based on the TG-latch circuit which is shown in Figure 9 and to synchronize the signals coming from the encoder. Master-slave flip-flops reduce the sensitivity to noise by minimizing the period of transparency. They operate on the clock front. The master-slave *D*-type flip-flop consists of 2 cascaded *D*-Latches in phase opposition. The first is called master; the second is called slave.  Figure 10 shows this circuit. It makes it possible to obtain a synchronous binary signal by means of the latch type flip-flops.

The operation of this circuit is as follows:

- The TG1 is in a conducting mode and transfers the data bit *D* to the stage one (master) latch, if the signal clock is in the state off (CLK = 0 ($\overset{-}{CLK}=1$)). The transfer of the data does not occur, when TG2 and TG3 are opened in the same time.
- When the signal clock is in the state on ($CLK=1\;\;(\overset{-}{CLK}=0$)), TG1 acts like a switch open and blocks changes in the data. In this time, TG2 switches off and completes the feedback latching circuit, while TG3 is off, to allow the data voltage to be passed into the stage two (slave) latch.

The *D* master-slave appears as a flip-flop having a data input *D* (data), a clock input (clock), and *Q* output. When the clock switches from the off state to the on state output *Q* is the value of *D* that has been presented which makes it a positive edge started storage element.

Logically, the operation of the TG-Register is as follows:

(2) $$\begin{array}{c} D\left(\;t\;\right)=Q\left(\;t+T\;\right). \end{array}$$

**Figure 11:**
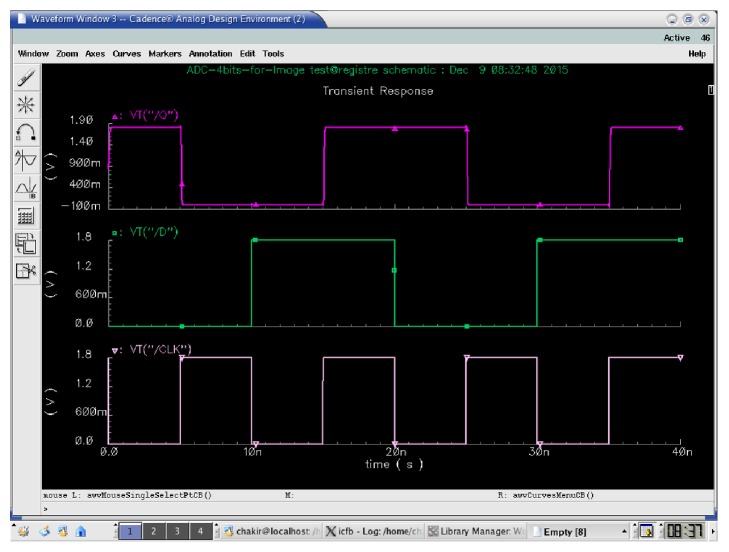
Simulation of the DFF.

The operation of a TG-Register is synchronous. Its role is to memorize a logical data at a precise moment. This data applied at *D* is taken into account at the beginning of the rising edge and transferred to the output *Q* at the end of this rising edge. A new transfer from input *D* to output *Q* will occur at the next rising edge of the clock.  Figure 11 shows the simulation of the TG-Register.

## 3. ADC Complete Simulation Results

**Figure 12:**
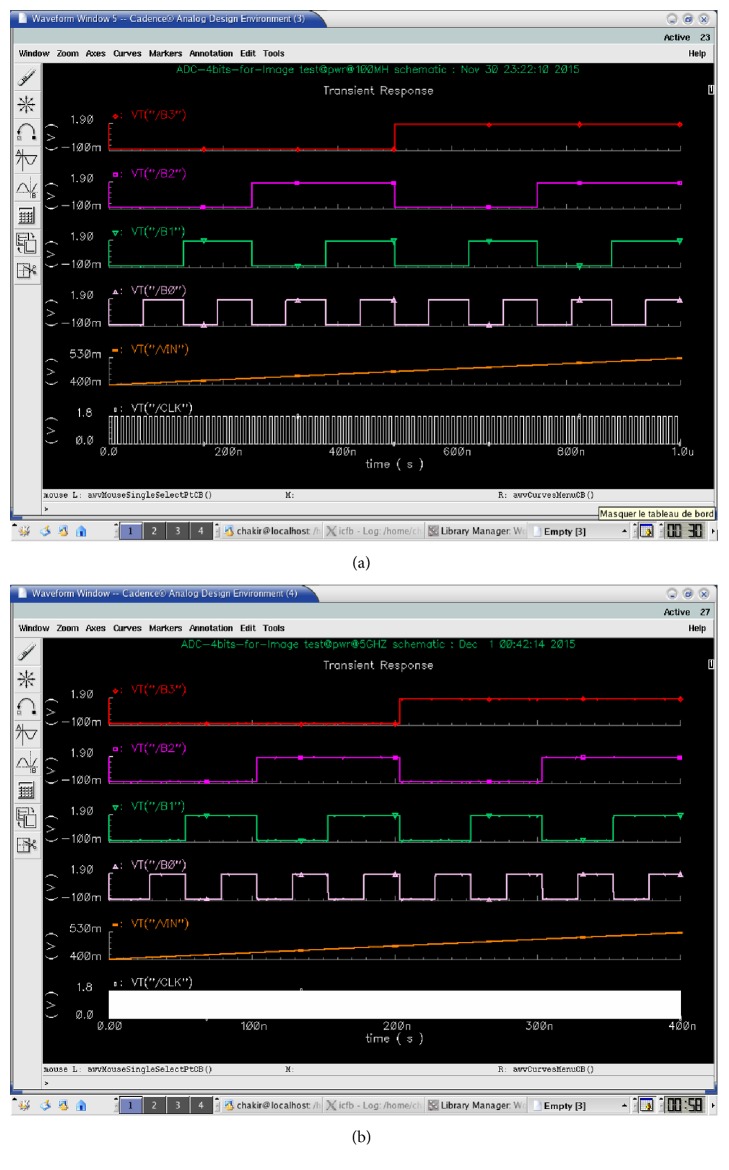
(a) A complete conversion of the ADC with a ramp input voltage of 125 mV@100 MHz. (b) A complete conversion of the ADC with a ramp input voltage of 125 mV@5 GHz.

**Figure 13:**
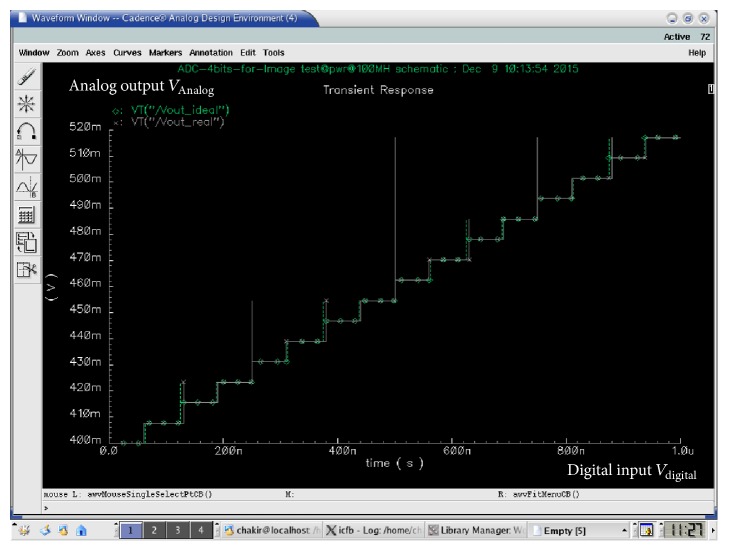
Transfer characteristic ideal and real of ADC in 100 MHz@125 mV.

**Figure 14:**
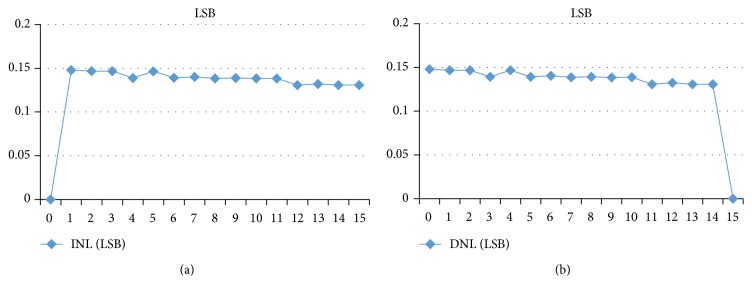
(a) INL error at 100 MHz. (b) DNL error at 100 MHz.

**Figure 15:**
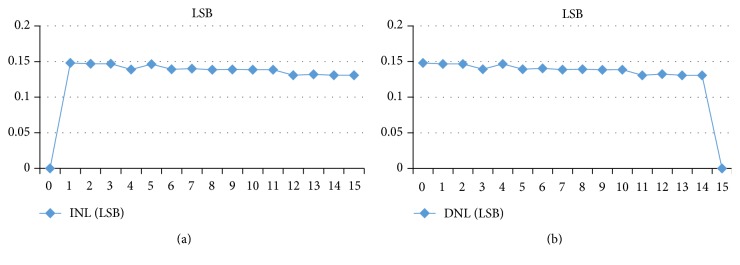
(a) INL error at 5 GHz. (b) DNL error at 5 GHz.

**Figure 16:**
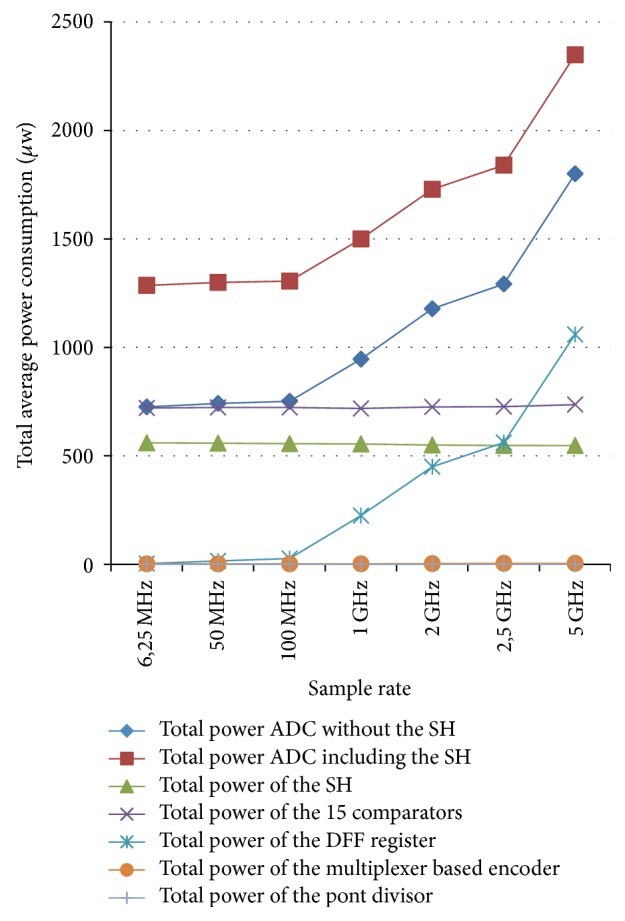
Average power consumption of subblocks ADC for varying frame rates with dynamic range of 125 mV.

The complete simulation of the proposed ADC is presented in Figures 12(a) and 12(b) for two-clock frequency 100 MHz (which represents 10 ns) and 5 GHz (which represents 200 ps). The low input MAPS sensor signal is 125 mV. This latter is a ramp that allows clarifying all the values encoded by the ADC. The supply voltage is 1.8 V in an ambient temperature of 27°C. Then, the outputs of the ADC are displayed in a signal from *B*_0_ to *B*_3_. We visually note that all binary values from 0000 to 1111 are presented and traversed in a homogeneous way on the duration of the simulation. For testing the output of the proposed ADC we will integrate an ideal DAC in the output of the realized ADC.  Figure 13 shows the transfer function of an ideal and real ADC, for a resolution of 4 bits. The horizontal axis represents the digital input *V*_digital_, and the vertical axis represents the analogy output *V*_analog_. The dynamics of the input bits *V*_digital_ are between 0000 and 1111. In the ideal case, the width and height of a “quantum” are constant and are, respectively, worth 1 LSB and VLSB. In reality, the function of the real transfer is altered by a number of parameters such as noise, the problems of matching between components, and the opening error of comparators. Indeed, these static errors can be described by only four parameters: the offset error, gain error, the DNL, and INL [41]. Figures 14(a)and 14(b) and 15(a) and 15(b) both, respectively, show the results of the differential nonlinearity (DNL) and the integral nonlinearity (INL) of ADC at the rate of 100 M samples/s and 5 G samples/s. The maximum DNL and INL are 0.0812/−0.0787 LSB and 0.0811/−0.0787 LSB, respectively, at the rate of 100 M samples/s and the maximum DNL and INL are 0.1480/0.1308 LSB and 0.1480/0.0000 LSB, respectively, at the rate of 5 G samples/s. The simulation results of the ADC are summarized in Table 4. The conversion time of the ADC is 10 ns at a sampling frequency of 100 MHz, realizing an integration time of 48 *μ*s for the full size sensor. Since the consumption is a determining factor in the design of our ADC we will study the variation of the ADC consummation according to the frequency, and more precisely we will calculate the consummation of each block of the ADC for different values of frequencies ranging from 6.25 MHz to 5 GHz.  Figure 16 shows the simulated average power consumption of subblocks ADC for varying frame rates with dynamic range of 125 mV. The average power consumption of the S/H and 15 comparators are between 560.09/547.93 *μ*W and 718.5/736.02 *μ*W, respectively, in a constant way for different sampling rates between 6.25 MHz and 5 GHz. The average power consumption of the multiplexor based encoder is between 1.67 and 3.92 *μ*W for different sampling rates (between 6.25 MHz and 5 GHz). The average power consumption of the pont divisor is 0.061 *μ*W constant for the same different sampling rates. The average power consumption of the register is between 3.17 and 1060 *μ*W; this value slightly rises to 100 MHz after being increased exponentially with increasing sampling rates. The proposed 4-bit column-parallel ADC Flash consumes less power 725.6 *μ*W and 1800 *μ*w without the S/H at a high speed sampling rate of 100 MS/s and of 5 GS/s, respectively; this value rises to 1.28 mW and 2.34 mW including the S/H at a high speed sampling rate of 100 MS/s and 5 GS/s, respectively.

**Figure 17:**
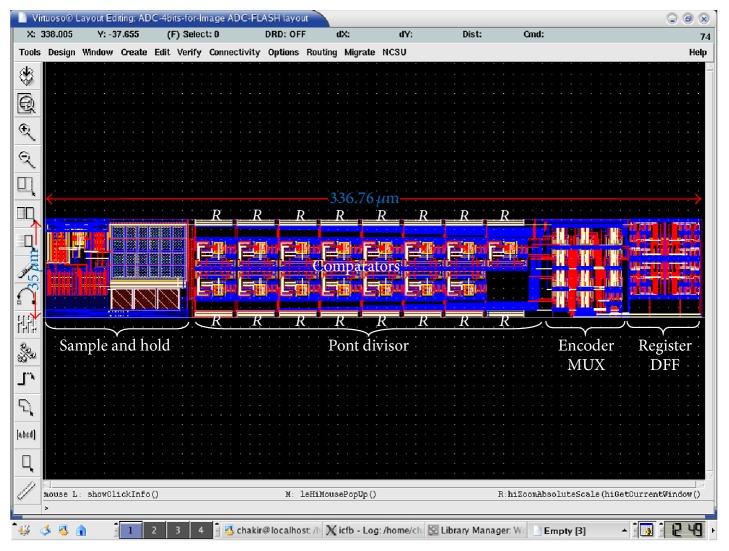
Layout of the proposed ADC.

The layout of our proposed ADC was realized in TSMC 0.18 *µ*m technology. It is quite difficult to quantify the difficulty of this design stage. To achieve drawing masks especially to not exceed 35 *µ*m width is to some extent a complicated thing. Thus, the lack of space for routing tracks, the lack of space for the placement of components, and the value of the parasitic capacitances associated with the form factor are just examples of the many challenges faced when drawing masks.  Figure 17 presented the drawing masks of proposed ADC, and its size area is 35 *µ*m × 336.76 *μ*m^2^.

### 3.1. Comparison and Discussion

The power and area efficiency of the proposed ADC have been compared to other works with different sampling rates and similar resolutions for Monolithic Active Pixel Sensor (MAPS). Table 4 shows the compared results of the proposed ADC with other state-of-the-art works ADCs [18, 20–22]. The pipeline ADC [20] can achieve a high speed, but it has larger power consumption. The ramp ADC [21] has a moderate sampling rate and it is satisfactory with the frame rate of MAPS for the VXD outer layers. Note that [18, 22] only includes static power consumption without the sample-and-hold circuit. Therefore, the power consumption has been compared with the other works. From the results, this ADC has one of the best power efficiencies of published work. Moreover, it achieves the lowest power consumption and a high speed more than 5 GHz. Also this ADC has the smallest active area of 35 × 336.76 *μ*m^2^.

## 4. Conclusion

In this paper, a new optimized architecture of a low power, high speed, and small-area 4-bit column-parallel ADC Flash integrated at the MAPS sensor array access per-column ADC (PC-ADC) has been proposed. To increase the sensitiveness of the converter to the very small amplitude of the input signal from the sensor and to provide a sufficient time to the converter to be able to code the input signal, we have proposed to interpose an optimized S/H block in the converter. The simulated results show that the architecture offers many interesting performances such as low power consumption of 751.42 *μ*W without the S/H at a high speed sampling rate of 100 MS/s; this value rises to 1.28 mW with the S/H. Its DNL and INL are 0.0812/−0.0787 LSB and 0.0811/−0.0787 LSB, respectively. Furthermore, this ADC achieves a high speed more than 5 GHz and has the smallest active area of 35 × 336.76 *μ*m^2^. Consequently, with these optimized characteristics, this kind of ADC can be used for monolithic active pixel sensors (MAPS) in high energy physics to accomplish the requirements for next generation with some GS/s.

## Figures and Tables

**Table 1:** Performance summary of the OTA.

Supply voltage	Gain	PM	GM	BW	SR	ICMR^−^	ICMR^+^	CMRR	PSRR^+^	PSRR^−^	Offset	Pd
1.8 V	103 dB	49° degree	11.46 dB	12.4 KHz	151.50 V/*µ*S	71.45 mV	1.60 mV	71.27 dB	73.60 dB	81.75 dB	11.30 mV	316.24 *µ*W

**Table 2:** Performance summary of the comparator.

Gain	SR	ICMR^−^	ICMR^+^	Offset	BW	Setting time	Pd
110.72 dB	3870 V/*µ*S	0 V	1.20 V	9 mV	17.17 MHz	2.28 ns	48 *µ*W

**Table 3:** The truth table for 4-bit multiplexer based encoder.

Thermometer code inputs *C*_14_ to *C*_0_															Binary codes *S*_3_ to *S*_0_				Estimated voltage (125 mV)
*C*_14_	*C*_13_	*C*_12_	*C*_11_	*C*_10_	*C*_9_	*C*_8_	*C*_7_	*C*_6_	*C*_5_	*C*_4_	*C*_3_	*C*_2_	*C*_1_	*C*_0_	*S*_3_	*S*_2_	*S*_1_	*S*_0_	
**0**	**0**	**0**	**0**	**0**	**0**	**0**	**0**	**0**	**0**	**0**	**0**	**0**	**0**	**0**	**0**	**0**	**0**	**0**	400–407.8125
**0**	**0**	**0**	**0**	**0**	**0**	**0**	**0**	**0**	**0**	**0**	**0**	**0**	**0**	1	**0**	**0**	**0**	1	407.8125–415.6250
**0**	**0**	**0**	**0**	**0**	**0**	**0**	**0**	**0**	**0**	**0**	**0**	**0**	1	1	**0**	**0**	1	**0**	415.6250–423.4375
**0**	**0**	**0**	**0**	**0**	**0**	**0**	**0**	**0**	**0**	**0**	**0**	1	1	1	**0**	**0**	1	1	423.4375–431.2500
**0**	**0**	**0**	**0**	**0**	**0**	**0**	**0**	**0**	**0**	**0**	1	1	1	1	**0**	1	**0**	**0**	431.2500–439.0625
**0**	**0**	**0**	**0**	**0**	**0**	**0**	**0**	**0**	**0**	1	1	1	1	1	**0**	1	**0**	1	439.0625–446.875
**0**	**0**	**0**	**0**	**0**	**0**	**0**	**0**	**0**	1	1	1	1	1	1	**0**	1	1	**0**	446.875–454.6875
**0**	**0**	**0**	**0**	**0**	**0**	**0**	**0**	1	1	1	1	1	1	1	**0**	1	1	1	454.6875–462.5000
**0**	**0**	**0**	**0**	**0**	**0**	**0**	1	1	1	1	1	1	1	1	1	**0**	**0**	**0**	462.5000–470.3125
**0**	**0**	**0**	**0**	**0**	**0**	1	1	1	1	1	1	1	1	1	1	**0**	**0**	1	470.3125–478.1250
**0**	**0**	**0**	**0**	**0**	1	1	1	1	1	1	1	1	1	1	1	**0**	1	**0**	478.1250–485.9375
**0**	**0**	**0**	**0**	1	1	1	1	1	1	1	1	1	1	1	1	**0**	1	1	485.9375–493.7500
**0**	**0**	**0**	1	1	1	1	1	1	1	1	1	1	1	1	1	1	**0**	**0**	493.7500–501.5625
**0**	**0**	1	1	1	1	1	1	1	1	1	1	1	1	1	1	1	**0**	1	501.5625–509.3750
**0**	1	1	1	1	1	1	1	1	1	1	1	1	1	1	1	1	1	**0**	509.3750–517.1875
1	1	1	1	1	1	1	1	1	1	1	1	1	1	1	1	1	1	1	517.1875–525

**Table 4:** Comparison to state-of-the-art works.

Parameters	[20]	[21]	[18]	[22]	This work	
Architecture	Pipelined^*∗*^	Ramp^*∗*^	SAR^*∗∗*^	SAR^*∗*^	Flash^*∗∗*^	
Technology (*µ*m)	0.35	0.35	0.35	0.35	0.18	
Supply voltage (V)	3.3 (Analog)	3.3	3	3	1.8	
Temperature (C°)	27	27	27	27	27	
Number of output bits	4	4	4/3/2	4/3/2	4	
Dynamic conversion (mV)	16	125/128	16	16	125	
LSB (mV)	1	7.81/7	1	1	7.81	
Sampling frequency	50 MS/s	1 MS/s	6.25 MS/s	6.25 MS/s	100 MS/s	5 GS/s
Dimension (*μ*m^2^)	80 × 900	25 × 900	35 × 545	35 × 545	35 × 336.76	
Power consumption						
*P*_ADC_^1^ (*µ*W)	—	744/—	714 (active)	714 (active)	751.42	1800
			486 (Inactive)	486 (Inactive)		
*P*_ADC_^2^ (*µ*W)	2600	—	—	—	1306	2348
INL (LSB)						
INL_MIN_	−0.41	—/−0.4	−0.15	−0.20	−0.0787	0.0000
INL_MAX_	0.69	—/1.0	0.05	0.29	0.0811	0.1480
DNL (LSB)						
DNL_MIN_	−0.4	—/−0.8	−0.09	−0.28	−0.0787	0.1308
DNL_MAX_	0.56	—/0.3	0.14	0.49	0.0812	0.1480
Gain error (LSB)	—	—	—	—	0.0411	0.1308
Offset error (LSB)	—	—	—	—	0.0387	0.1480

^*∗*^Testing results. ^*∗∗*^Simulation results.

^1^The total power consumption of the ADC, without the power consumption of the S/H.

^2^The total power consumption of the ADC, including the power consumption of the S/H.
